# Supplementary material for: Neuronal hyperactivity in neurons derived from individuals with gray matter heterotopia
Source: Nat Commun. 2025 Feb 18;16:1737. doi: 10.1038/s41467-025-56998-1 (PMC11836124; doi:10.1038/s41467-025-56998-1)
Supplement: Supplementary file 1 — Supplementary Information [file 41467_2025_56998_MOESM1_ESM.pdf]

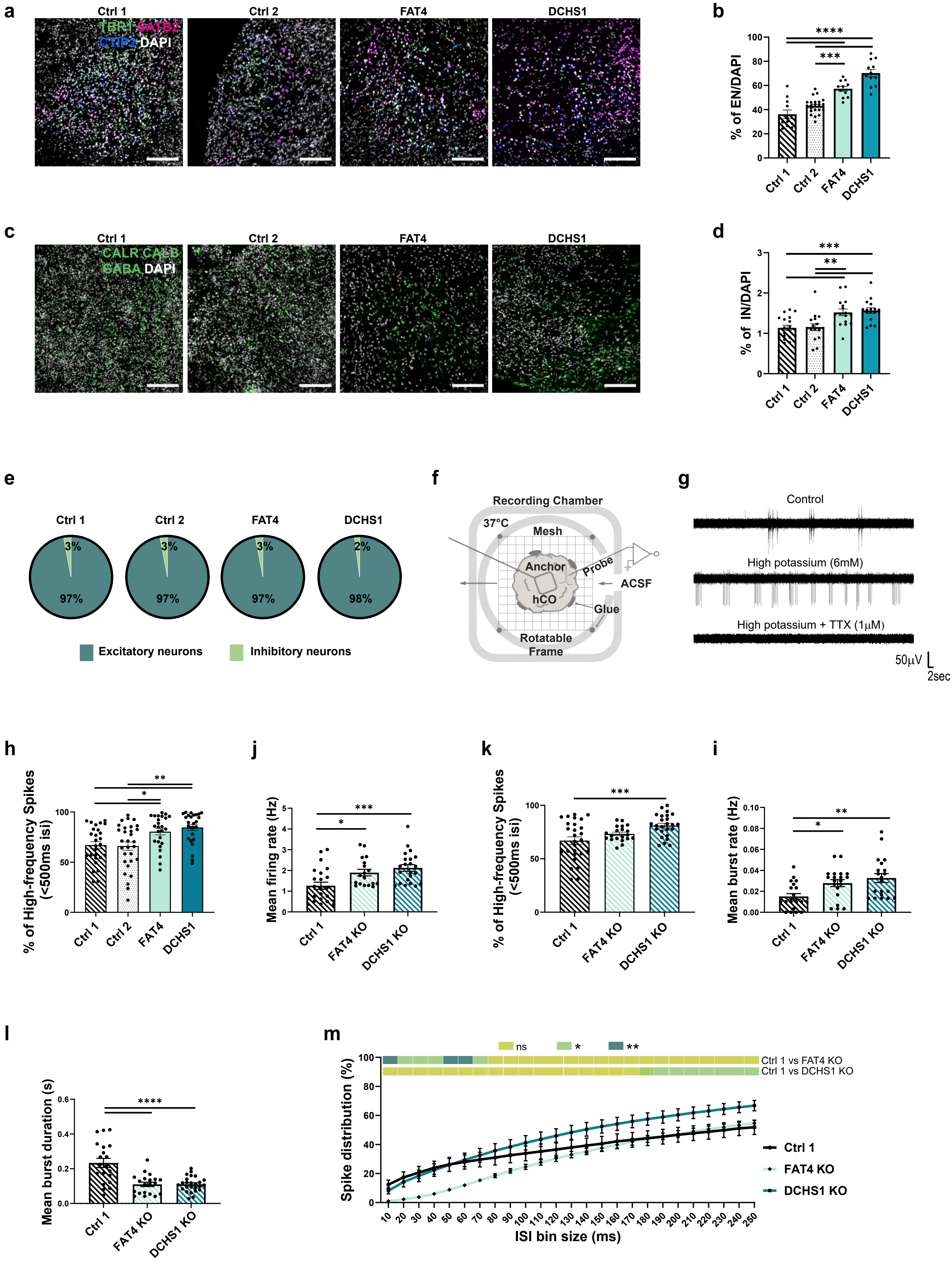
a
Ctrl 1
Ctrl 2
FAT4
DCHS1
TBR1 SATB2
CTIP2 DAPI
b
% of EN/DAPI
****
***
Ctrl 1
Ctrl 2
FAT4
DCHS1
c
CALR CALB
GABA DAPI
d
% of IN/DAPI
***
**
e
3%
97%
2%
98%
Excitatory neurons
Inhibitory neurons
f
Recording Chamber
37°C
Mesh
Anchor
Probe
ACSF
hCO
Glue
Rotatable
Frame
g
Control
High potassium (6mM)
High potassium + TTX (1μM)
50μV
2sec
h
% of High-frequency Spikes
(<500ms isi)
j
Mean firing rate (Hz)
FAT4 KO
DCHS1 KO
k
i
Mean burst rate (Hz)
l
Mean burst duration (s)
****
m
ns
Ctrl 1 vs FAT4 KO
Ctrl 1 vs DCHS1 KO
Spike distribution (%)
ISI bin size (ms)
Ctrl 1
FAT4 KO
DCHS1 KO

Supplementary Figure 1. Characterization of control-, patient-derived and KO hCOs

a-d. Micrographs of 9 months old hCOs sections immunostained for excitatory neuron (EN) (TBR1, SATB2, CTIP2) (a) and inhibitory neuron (IN) (CALR, CALB, GABA) markers (c) and quantifications of the percentage of positive cells/DAPI (b,d)

e. Quantification of the ratio of EN and IN in control- and patient-derived hCOs.

f. Detailed scheme of silicon probe recording in intact hCOs. ACSF (artificial cerebral spinal fluid).

g. Representative recording traces of spontaneous spike activity in a hCO under different pharmacological conditions (Control, High potassium and High potassium + Tetrodotoxin (TTX)). Recordings were performed for 30 sec.

h. Quantification of the percentage of high-frequency spikes (<500 ms ISI) recorded in control- and patient-derived hCOs.

j-l. Quantification of the mean firing rate (j), high frequency spikes (k), mean burst rate (i) and mean burst duration (l) recorded in control- and KO hCOs.

m. Quantification of the spike distribution obtained for control-derived and KO hCOs.

Scale bars: 50 µm (a, c). Data are represented as mean ± SEM. Statistical significance was based on one-way (b, d, h-l) and two-way (m) ANOVA with Turkey's multiple comparison tests (*P < 0.05, **P < 0.01, ***P < 0.001, ****P < 0.0001). Independent hCOs (b, d, h-m) were analyzed. Every dot in the plots refers to independent field of view (b,d) or independently analyzed recording areas (h-l). At least eleven (n=11) randomly chosen fields of view or eighteen (n=18) recording areas were analyzed across three independent batches (N=3). Source data are provided as a Source Data file, including the exact p-values and n numbers.

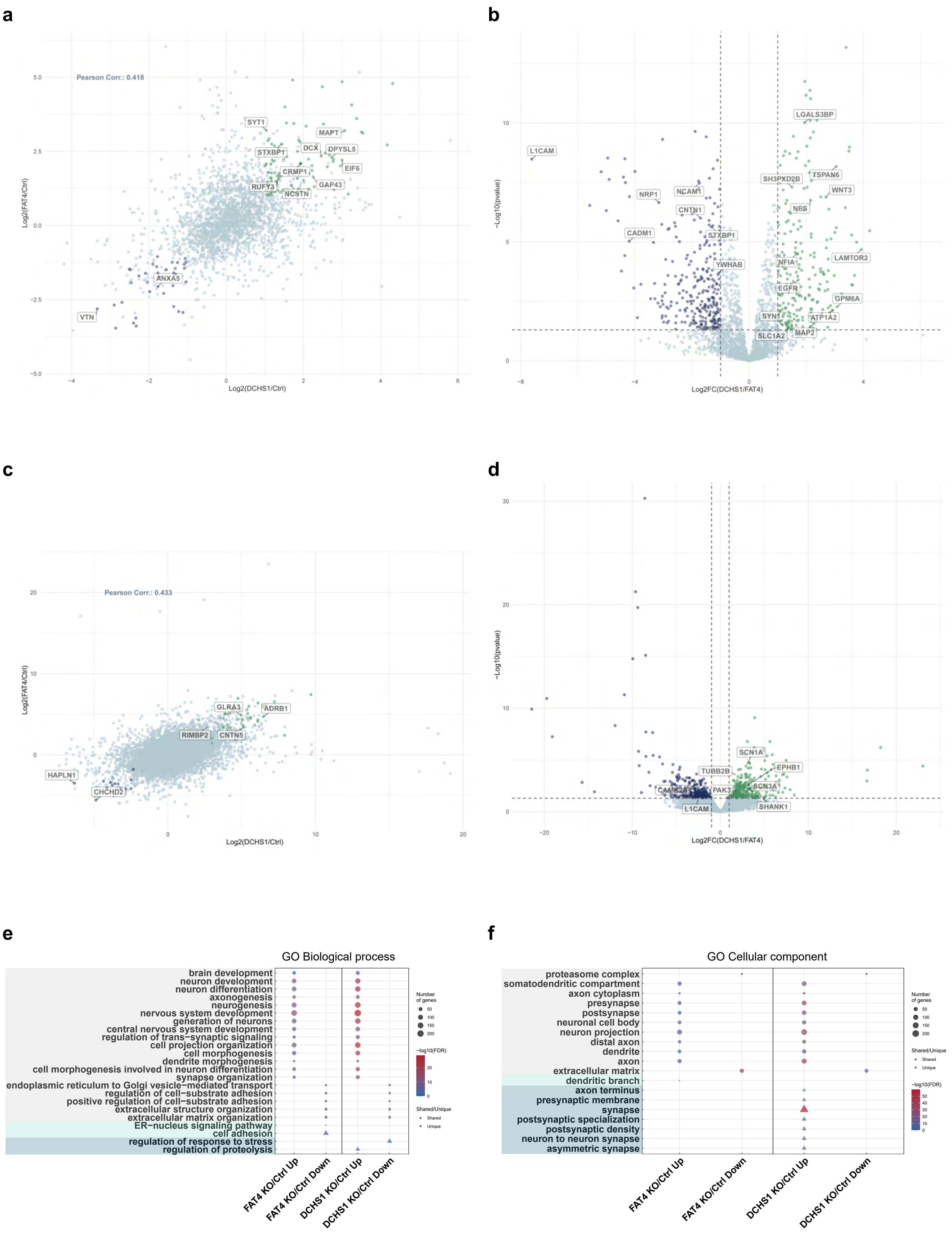
a
Pearson Corr.: 0.418
SYT1
MAPT
STXBP1
DCX
DPYSL5
EIF6
CRMP1
RUFY3
GAP43
NCSTN
ANXA5
VTN
Log2(FAT4/Ctrl)
Log2(DCHS1/Ctrl)
b
LGALS3BP
L1CAM
SH3PXD2B
TSPAN6
NCAM1
WNT3
NRP1
CNTN1
NES
CADM1
STXBP1
YWHAB
NFIA
LAMTOR2
EGFR
GPM6A
SYN1
ATP1A2
SLC1A2
MAP2
−Log10(pvalue)
Log2FC(DCHS1/FAT4)
c
Pearson Corr.: 0.433
GLRA3
ADRB1
RIMBP2
CNTN5
HAPLN1
CHCHD2
Log2(FAT4/Ctrl)
Log2(DCHS1/Ctrl)
d
SCN1A
EPHB1
TUBB2B
CAMK2A
PAK3
SCN3A
L1CAM
SHANK1
−Log10(pvalue)
Log2FC(DCHS1/FAT4)
e
GO Biological process
brain development
neuron development
neuron differentiation
axonogenesis
neurogenesis
nervous system development
generation of neurons
central nervous system development
regulation of trans−synaptic signaling
cell projection organization
cell morphogenesis
dendrite morphogenesis
cell morphogenesis involved in neuron differentiation
synapse organization
endoplasmic reticulum to Golgi vesicle−mediated transport
regulation of cell−substrate adhesion
positive regulation of cell−substrate adhesion
extracellular structure organization
extracellular matrix organization
ER−nucleus signaling pathway
cell adhesion
regulation of response to stress
regulation of proteolysis
Number of genes
−log10(FDR)
Shared/Unique
Shared
Unique
FAT4 KO/Ctrl Up
FAT4 KO/Ctrl Down
DCHS1 KO/Ctrl Up
DCHS1 KO/Ctrl Down
f
GO Cellular component
proteasome complex
somatodendritic compartment
axon cytoplasm
presynapse
postsynapse
neuronal cell body
neuron projection
distal axon
dendrite
axon
extracellular matrix
dendritic branch
axon terminus
presynaptic membrane
synapse
postsynaptic specialization
postsynaptic density
neuron to neuron synapse
asymmetric synapse
Number of genes
Shared/Unique
Shared
Unique
−log10(FDR)
FAT4 KO/Ctrl Up
FAT4 KO/Ctrl Down
DCHS1 KO/Ctrl Up
DCHS1 KO/Ctrl Down

g

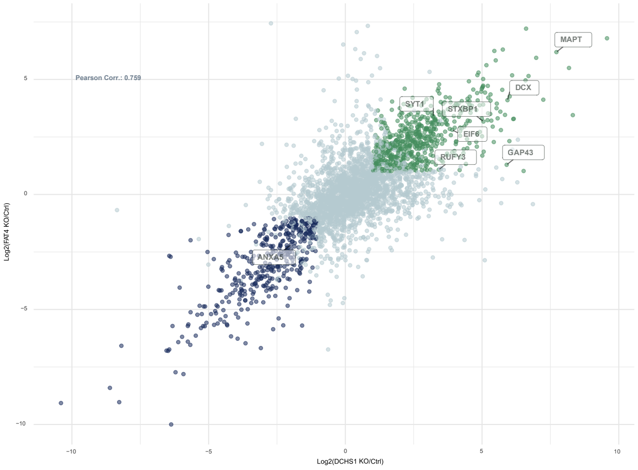
Pearson Corr.: 0.759
MAPT
DCX
SYT1
STXBP1
EIF6
GAP43
RUFY3
ANXA5
Log2(FAT4 KO/Ctrl)
Log2(DCHS1 KO/Ctrl)
5
0
−5
−10
−10
−5
0
5
10

h

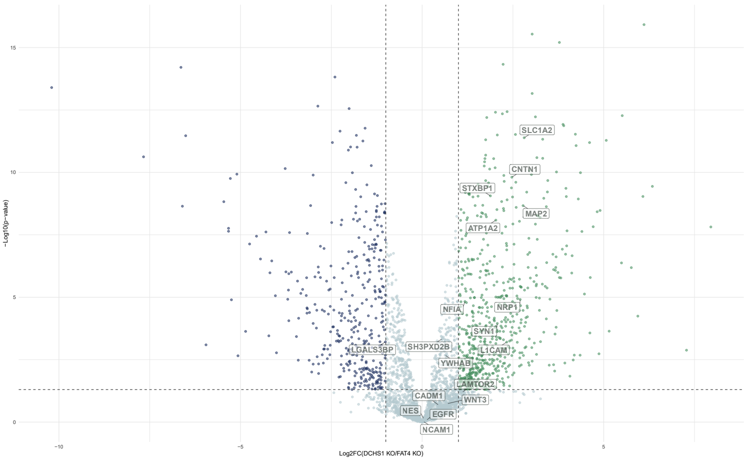
SLC1A2
CNTN1
STXBP1
MAP2
ATP1A2
NFIA
NRP1
SYN1
SH3PXD2B
LGALS3BP
L1CAM
YWHAB
LAMTOR2
CADM1
WNT3
NES
EGFR
NCAM1
−Log10(p−value)
Log2FC(DCHS1 KO/FAT4 KO)
15
10
5
0
−10
−5
0
5

Supplementary Figure 2. Transcriptome and proteome analysis of control- and patient-derived and KO hCOs.

a. Pearson correlation analysis indicating proteins dysregulated with the same trend in the patient' conditions related to controls. Proteins upregulated are displayed in green, downregulated proteins in blue.

b. Volcano plot of dysregulated proteins between FAT4 and DCHS1 hCOs (Supplementary data 1). Proteins upregulated in DCHS1 hCOs related to FAT4 are displayed in green, downregulated proteins in blue.

c. Pearson correlation analysis indicating genes dysregulated with the same trend in the patient' conditions related to controls. Upregulated genes are displayed in green, downregulated genes in blue.

d. Volcano plot of dysregulated genes between FAT4 and DCHS1 neurons (Supplementary data 2). Genes upregulated in DCHS1 neurons are displayed in green, downregulated genes in blue.

e-f. Biological processes (e) and cellular components (f) enriched by GO analysis of the proteome data (Supplementary data 1). Common and FAT4 KO- or DCHS1 KO-specific enrichments are highlighted.

g. Pearson correlation analysis indicating proteins dysregulated with the same trend in the KO conditions related to controls. Proteins upregulated are displayed in green, downregulated proteins in blue.

h. Volcano plot of dysregulated proteins between FAT4 KO and DCHS1 KO hCOs (Supplementary data 1). Proteins upregulated in DCHS1 KO hCOs related to FAT4 KO are displayed in green, downregulated proteins in blue.

The x-axis represents the log2-fold change in abundance and y-axis the -log10 (p value).

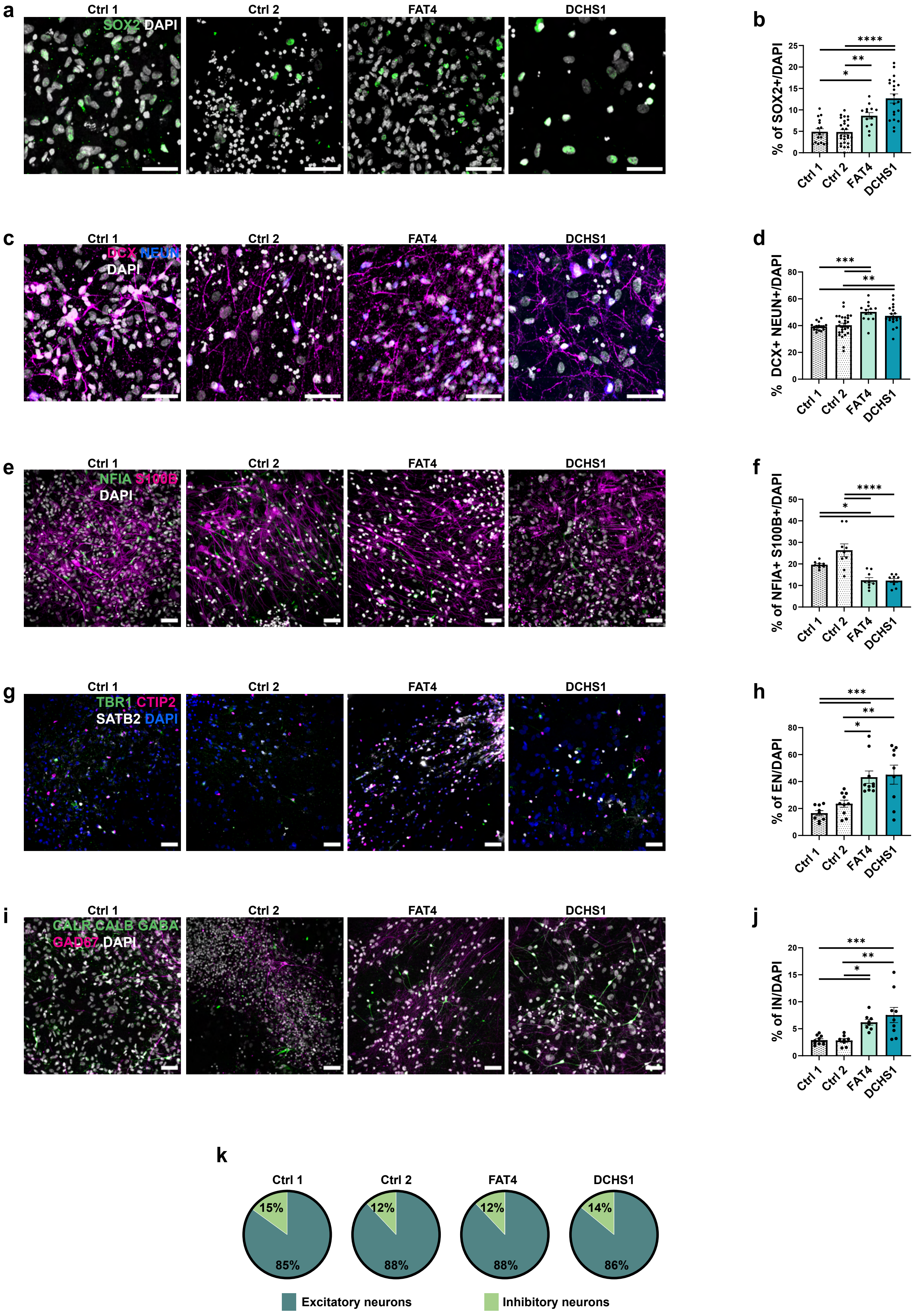
a
Ctrl 1
Ctrl 2
FAT4
DCHS1
SOX2 DAPI
b
% of SOX2+/DAPI
25
20
15
10
5
0
Ctrl 1
Ctrl 2
FAT4
DCHS1
****
**
*
c
Ctrl 1
Ctrl 2
FAT4
DCHS1
DCX NEUN
DAPI
d
% DCX+ NEUN+/DAPI
80
60
40
20
0
***
**
Ctrl 1
Ctrl 2
FAT4
DCHS1
e
Ctrl 1
Ctrl 2
FAT4
DCHS1
NFIA S100B
DAPI
f
% of NFIA+ S100B+/DAPI
50
40
30
20
10
0
****
*
Ctrl 1
Ctrl 2
FAT4
DCHS1
g
Ctrl 1
Ctrl 2
FAT4
DCHS1
TBR1 CTIP2
SATB2 DAPI
h
% of EN/DAPI
80
60
40
20
0
***
**
*
Ctrl 1
Ctrl 2
FAT4
DCHS1
i
Ctrl 1
Ctrl 2
FAT4
DCHS1
CALR CALB GABA
GAD67 DAPI
j
% of IN/DAPI
20
15
10
5
0
***
**
*
Ctrl 1
Ctrl 2
FAT4
DCHS1
k
Ctrl 1
Ctrl 2
FAT4
DCHS1
15%
85%
12%
88%
12%
88%
14%
86%
Excitatory neurons
Inhibitory neurons

l

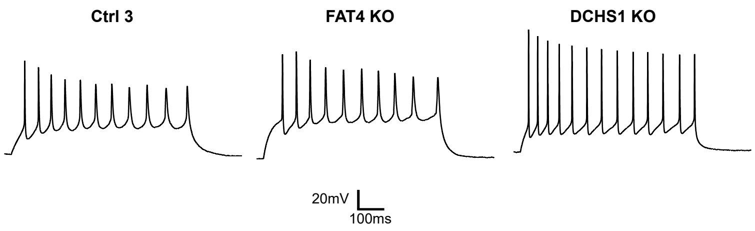

Ctrl 3
FAT4 KO
DCHS1 KO
20mV
100ms

m

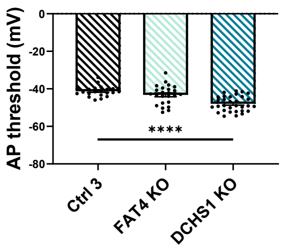

AP threshold (mV)
0
-20
-40
-60
-80
****
Ctrl 3
FAT4 KO
DCHS1 KO

n

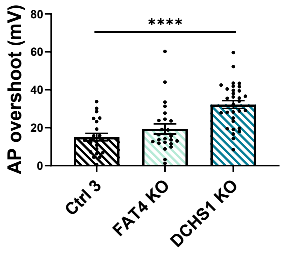

AP overshoot (mV)
80
60
40
20
0
****
Ctrl 3
FAT4 KO
DCHS1 KO

o

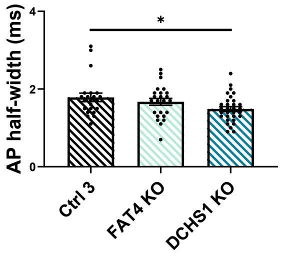

AP half-width (ms)
4
2
0
*
Ctrl 3
FAT4 KO
DCHS1 KO

p

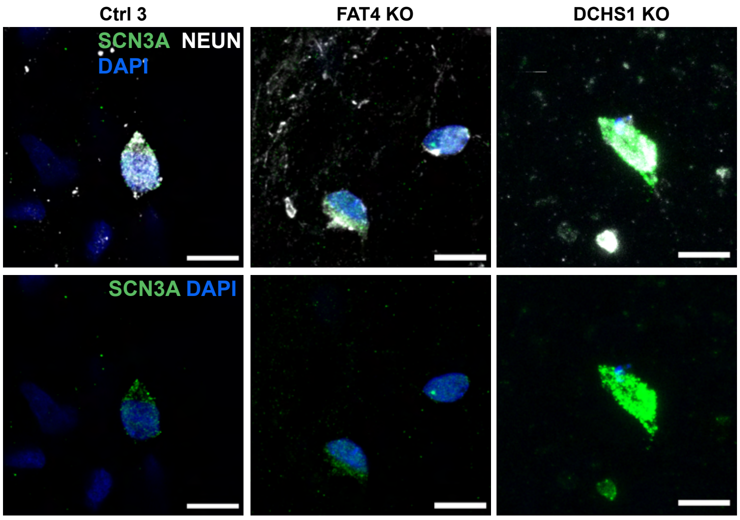

Ctrl 3
FAT4 KO
DCHS1 KO
SCN3A NEUN
DAPI
SCN3A DAPI

q

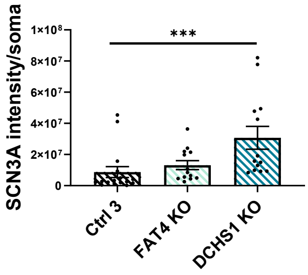

SCN3A intensity/soma
1×10⁸
8×10⁷
6×10⁷
4×10⁷
2×10⁷
0
***
Ctrl 3
FAT4 KO
DCHS1 KO

r

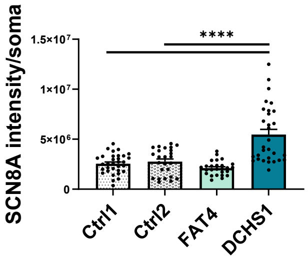

SCN8A intensity/soma
1.5×10⁷
1×10⁷
5×10⁶
0
****
Ctrl1
Ctrl2
FAT4
DCHS1

s

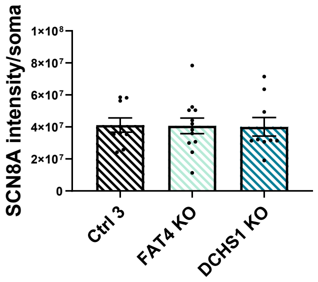

SCN8A intensity/soma
1×10⁸
8×10⁷
6×10⁷
4×10⁷
2×10⁷
0
Ctrl 3
FAT4 KO
DCHS1 KO

Supplementary Figure 3. Characterization of 2D neuronal cultures and investigation of single-cell electrophysiological properties and somatic VGSC densities in control-, patient-derived and KO neuronal cultures.

a-j. Micrographs of 10 weeks old control- and patient-derived 2D neurons immunostained for progenitor (SOX2) (a), neuronal (DCX, NEUN) (c), glial (NFIA, S100B) (e), excitatory neuron (EN) (TBR1, SATB2, CTIP2) (g) and inhibitory neuron (IN) (CALR, CALB, GABA, GAD67) (i) markers and quantifications of the percentage of positive cells/DAPI (b, d, f, h, j).

k. Quantification of the ratio of EN and IN in control- and patient-derived 2D neurons.

l. Representative patch-clamp recording traces depicting evoked neuronal firing of 10 weeks old control-derived and KO neurons. Recordings were performed in current-clamp mode.

m-o. Quantification of the AP threshold (m), AP overshoot (n) and AP half-width (o) for control-derived and KO neurons.

p-q. Micrographs of 10 weeks old control-derived and KO 2D neurons immunostained for NEUN and SCN3A (p) and quantification of the somatic SCN3A intensity (q).

r-s. Quantification of the somatic SCN8A intensity of control-, patient-derived (r) and KO (s) 2D neurons.

Scale bars: 50 µm (a, c, e, g, i), 10 µm (p). Data are represented as mean ± SEM. Statistical significance was based on one-way ANOVA with Turkey's multiple comparison tests (*P < 0.05, **P < 0.01, ***P < 0.001, ****P < 0.0001). Independent wells (b, d, f, h, j, m-o, q-s) were analyzed. Every dot in the plots refers to independent field of view (b, d, f, h, j) or independently analyzed neurons (m-o, q-s). At least eight (n=8) randomly chosen fields of view or nine (n=9) neurons were analyzed across three independent batches (N=3). Source data are provided as a Source Data file, including the exact p-values and n numbers.

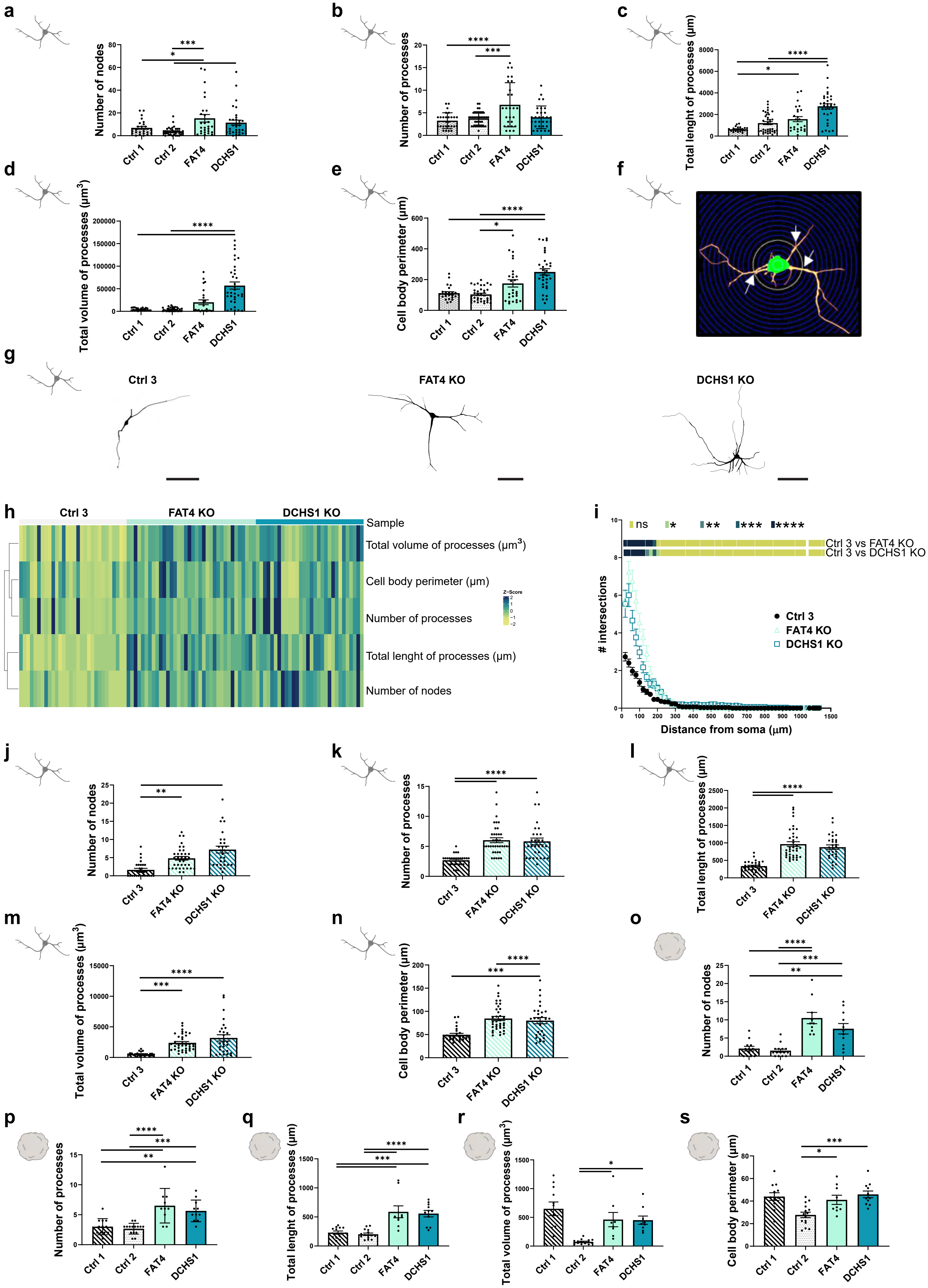

a
Number of nodes
Ctrl 1
Ctrl 2
FAT4
DCHS1
b
Number of processes
c
Total lenght of processes (µm)
d
Total volume of processes (µm³)
e
Cell body perimeter (µm)
f
g
Ctrl 3
FAT4 KO
DCHS1 KO
h
Sample
Total volume of processes (µm³)
Cell body perimeter (µm)
Number of processes
Total lenght of processes (µm)
Number of nodes
Z-Score
i
ns
Ctrl 3 vs FAT4 KO
Ctrl 3 vs DCHS1 KO
intersections
Distance from soma (µm)
j
k
l
m
n
o
p
q
r
s

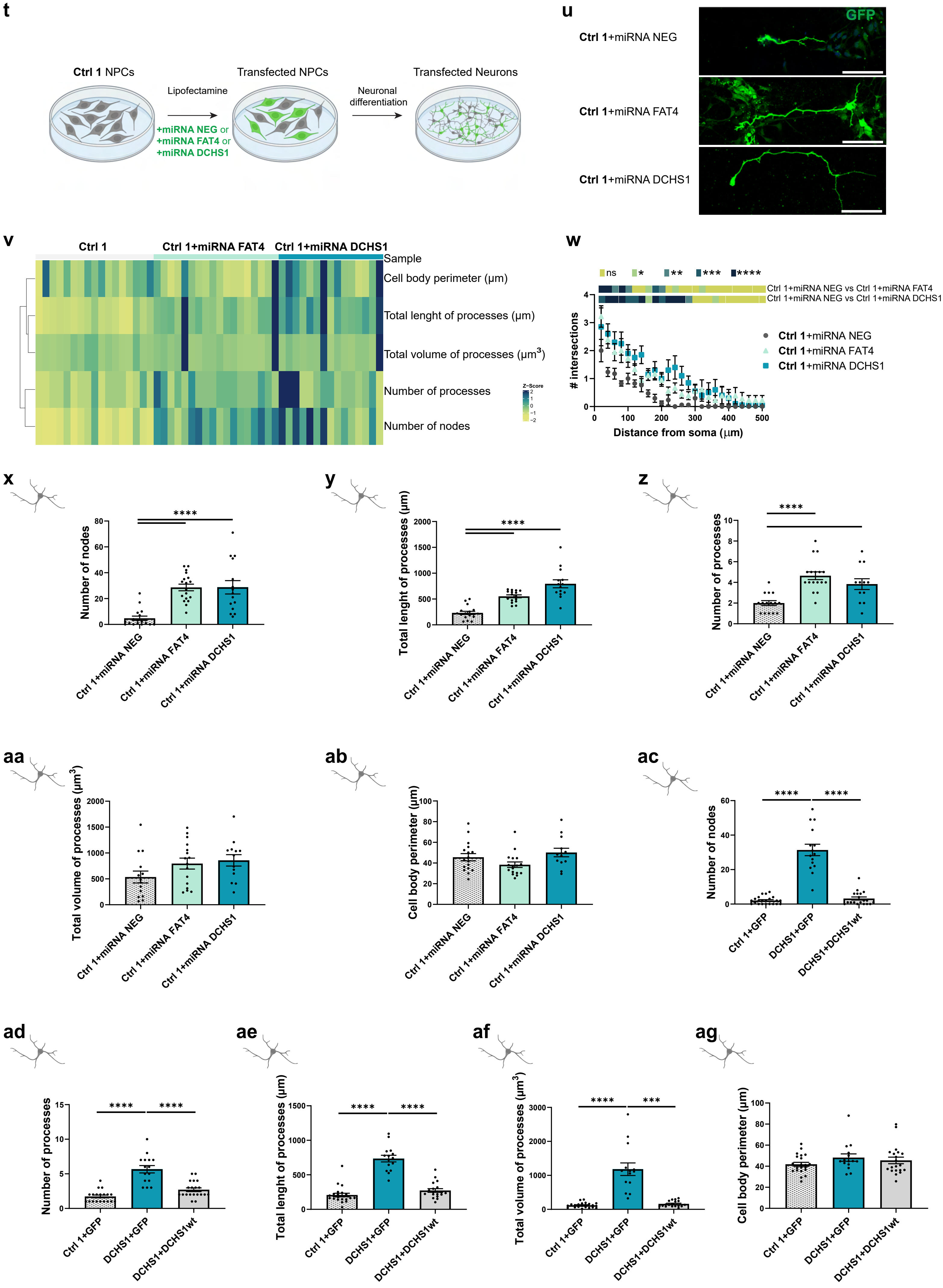
t
Ctrl 1 NPCs
Lipofectamine
+miRNA NEG or
+miRNA FAT4 or
+miRNA DCHS1
Transfected NPCs
Neuronal differentiation
Transfected Neurons
u
Ctrl 1+miRNA NEG
Ctrl 1+miRNA FAT4
Ctrl 1+miRNA DCHS1
GFP
v
Ctrl 1
Ctrl 1+miRNA FAT4
Ctrl 1+miRNA DCHS1
Sample
Cell body perimeter (µm)
Total lenght of processes (µm)
Total volume of processes (µm3)
Number of processes
Number of nodes
Z-Score
w
ns
*
**
***
****
Ctrl 1+miRNA NEG vs Ctrl 1+miRNA FAT4
Ctrl 1+miRNA NEG vs Ctrl 1+miRNA DCHS1
intersections
Distance from soma (µm)
Ctrl 1+miRNA NEG
Ctrl 1+miRNA FAT4
Ctrl 1+miRNA DCHS1
x
Number of nodes
****
Ctrl 1+miRNA NEG
Ctrl 1+miRNA FAT4
Ctrl 1+miRNA DCHS1
y
Total lenght of processes (µm)
****
z
Number of processes
****
aa
Total volume of processes (µm3)
ab
Cell body perimeter (µm)
ac
Number of nodes
**** ****
Ctrl 1+GFP
DCHS1+GFP
DCHS1+DCHS1wt
ad
Number of processes
**** ****
ae
Total lenght of processes (µm)
**** ****
af
Total volume of processes (µm3)
**** ***
ag
Cell body perimeter (µm)

Supplementary Figure 4. Morphological analysis of control-, patient-derived and KO neurons

a-e. Quantification of the number of nodes (a), number of processes (b), length of processes (c), volume of processes (d) and cell body perimeter (e) of reconstructed 10 weeks old control- and patient-derived 2D neurons.

f. Scheme of Sholl analysis. White arrows show intersections at a certain distance from the soma.

g. Morphologies of representative 10 weeks old control-derived and KO 2D neurons reconstructed with Neurolucida software.

h. Heatmap of the morphological characterization of reconstructed KO 2D neurons. Z-scores of analyzed parameters are displayed as colors ranging from yellow to blue as shown in the key.

i. Quantification of the number of intersections related to the distance from the soma of control-derived and KO 2D neurons, obtained by Sholl analysis.

j-n. Quantification of the number of nodes (j), number of processes (k), length of processes (l), volume of processes (m) and cell body perimeter (n) of reconstructed 10 weeks old control-derived and KO 2D neurons.

o-s. Quantification of the number of nodes (o), number of processes (p), length of processes (q), volume of processes (r) and cell body perimeter (s) of reconstructed 9 months old control- and patient-derived 3D neurons.

t. Scheme of the 2D neuronal differentiation of control NPCs upon FAT4 and DCHS1 KD. Scheme partially created with BioRender.com

u. Micrographs of 7 days old control neurons (9dpt) immunostained for GFP.

v. Heatmap of the morphological characterization of control neurons upon FAT4 and DCHS1 KD. pEGFP vector was used as control. Z-scores of analyzed parameters are displayed as colors ranging from yellow to blue as shown in the key.

w. Quantification of the number of intersections related to the distance from the soma of 7 days old control neurons (9dpt) upon FAT4 and DCHS1 KD, obtained by Sholl analysis.

x-ab. Quantification of the number of nodes (x), number of processes (y), length of processes (z), volume of processes (aa) and cell body perimeter (ab) of reconstructed 7 days old control neurons (9dpt) upon FAT4 and DCHS1 KD.

ac-ag. Quantification of the number of nodes (ac), number of processes (ad), length of processes (ae), volume of processes (af) and cell body perimeter (ag) of reconstructed 7 days old DCHS1 neurons (9dpt) upon *DCHS1*-wt expression.

Scale bars: 100 µm. Data are represented as mean ± SEM. Statistical significance was based on one-way (a-e; j-s; x-ag) and two-way (i, w) ANOVA with Turkey's multiple comparison tests (a-e; j-s; x-ag) and on Fisher's Exact (h, v) (*P < 0.05, **P < 0.01, ***P < 0.001, ****P < 0.0001). Independent wells (a-e, i-n, w-ag) or hCOs (o-s) were analyzed. Every dot in the plots refers to independently analyzed neurons (a-e, j-s, x-ag). At least eight (n=8) randomly chosen neurons were analyzed across three independent batches (N=3). Source data are provided as a Source Data file, including the exact p-values and n numbers. *Created in BioRender. Di Matteo, F. (2025) https://BioRender.com/m95d251, https://BioRender.com/v53q942*

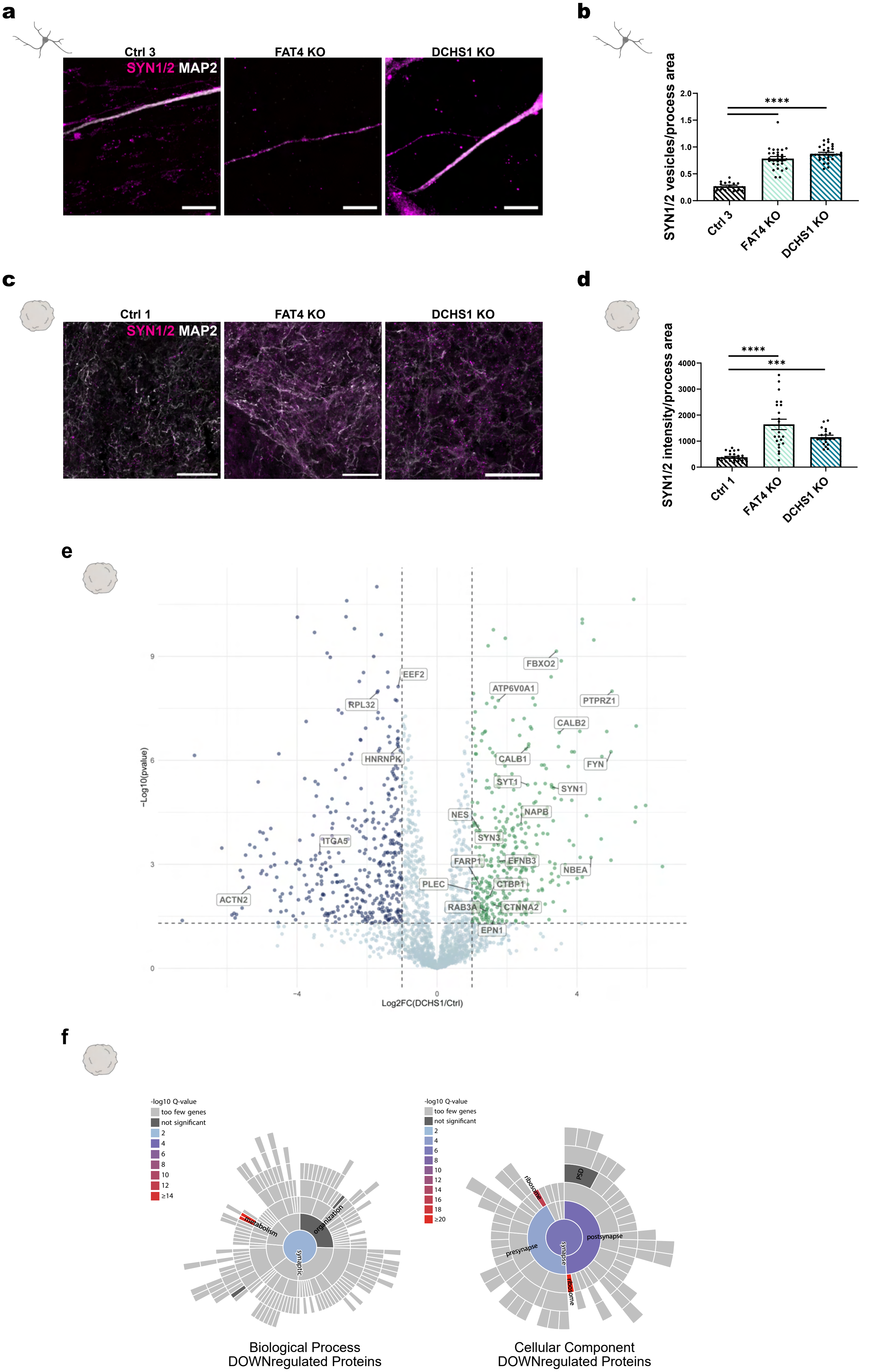

a
Ctrl 3
FAT4 KO
DCHS1 KO
SYN1/2 MAP2
b
SYN1/2 vesicles/process area
2.0
1.5
1.0
0.5
0.0
****
Ctrl 3
FAT4 KO
DCHS1 KO
c
Ctrl 1
FAT4 KO
DCHS1 KO
SYN1/2 MAP2
d
SYN1/2 intensity/process area
4000
3000
2000
1000
0
****
***
Ctrl 1
FAT4 KO
DCHS1 KO
e
-Log10(pvalue)
Log2FC(DCHS1/Ctrl)
9
6
3
0
-4
0
4
FBXO2
EEF2
ATP6V0A1
RPL32
PTPRZ1
CALB2
HNRNPK
CALB1
FYN
SYT1
SYN1
NES
NAPB
ITGA5
SYN3
FARP1
EFNB3
NBEA
PLEC
CTBP1
ACTN2
RAB3A
CTNNA2
EPN1
f
-log10 Q-value
too few genes
not significant
2
4
6
8
10
12
≥14
metabolism
organization
synaptic
Biological Process
DOWNregulated Proteins
-log10 Q-value
too few genes
not significant
2
4
6
8
10
12
14
16
18
≥20
PSD
ribosome
presynapse
postsynapse
synapse
ribosome
Cellular Component
DOWNregulated Proteins

Supplementary Figure 5. Investigation of synaptic properties in control-, patient-derived and KO neuronal cultures.

a-b. Micrographs of 10 weeks old control-derived and KO 2D neurons immunostained for MAP2 and SYN1/2 (a) and quantification (b) of the number of SYN1/2 puncta related to the MAP2 process area of control-derived and KO neurons.

c-d. Micrographs of sections of 9 months old control-derived and KO hCOs immunostained for MAP2 and SYN1/2 (c) and quantification (d) of the intensity of SYN1/2 puncta related to the MAP2 process area of control-derived and KO neurons.

e. Volcano plot of dysregulated proteins between control and DCHS1 synaptosomes enriched fractions (Supplementary data 1). The x-axis represents the log2-fold change in abundance and y-axis the -log10 (p value). Proteins upregulated in DCHS1 neurons related to control are displayed in green, the downregulated in blue.

f. Graph showing significantly-enriched GO terms of the proteome analysis performed on fractions enriched in synaptosomes, isolated from hCOs. GO analysis shows enrichment for biological processes and cellular components of proteins downregulated in DCHS1 synaptosomes (Supplementary data 1).

Scale bars: 10 µm (a), 50 µm (c). Data are represented as mean ± SEM. Statistical significance was based on one-way ANOVA with Turkey's multiple comparison tests (b, d) and Fisher's Exact (e, f) (***P < 0.001, ****P < 0.0001). Independent wells (b) or hCOs (d) were analyzed. Every dot in the plots refers to independently analyzed neurons (b, d). At least fifteen (n=15) randomly chosen neuronal processes were analyzed across three independent batches (N=3). Source data are provided as a Source Data file, including the exact p-values and n numbers. *Created in BioRender. Di Matteo, F. (2025)* [*https://BioRender.com/m95d251*](https://BioRender.com/m95d251)

	Ctrl 1 (n = 37)	Ctrl 2 (n = 23)	FAT4 (n = 18)	p1	Δ1	p2	Δ2
V_{rest} (mV)	-60,5 ± 1,0	-55,3 ± 0,6	-57,5 ± 1,6	NS		NS	
C_N (pF)	49,6 ± 2,5	44,2 ± 1,5	43,5 ± 3,8	NS		NS	
AP threshold (mV)	-39,7 ± 0,9	-39,9 ± 0,6	-40,0 ± 1,8	NS		NS	
AP overshoot (mV)	11,9 ± 1,1	14,3 ± 1,2	10,4 ± 1,5	NS		NS	
AP half-width (ms)	1,8 ± 0,05	1,7 ± 0,03	1,9 ± 0,07	NS		NS	
fAHP (mV)	-20,8 ± 1,0	-19,3 ± 0,7	-18,2 ± 1,7	NS		NS	

Supplementary Table 1. Investigation of single-cell electrophysiological properties in control- and FAT4-derived neuronal cultures.

Quantification of patch-clamp recordings of 10 weeks old control- and FAT4-derived neurons. Recordings were performed in current-clamp mode. Data are represented as mean ± SEM. Statistical significance was based on one-way ANOVA with Turkey's multiple comparison tests. Independent wells were analyzed. At least eighteen (n=18) randomly chosen neurons were analyzed across three independent batches (N=3). The exact p-values are not indicated as there are no significant differences, the n numbers are shown in the table. Source data are provided as a Source Data file for the parameters significantly different, which graphs are shown in the Fig. 3d-f.

	Ctrl 3 (n = 22)	FAT4 KO (n = 24)	DCHS1 KO (n = 31)	p1	Δ1	p2	Δ2
V_{rest} (mV)	-60,7 ± 0,8	-56,7 ± 0,8	-60,2 ± 0,8	NS		NS	
C_N (pF)	46,1 ± 3,9	53,6 ± 2,9	60,9 ± 4,2	NS		NS	
**AP threshold (mV)**	**-41,3 ± 0,4**	-43,2 ± 0,6	**-48,0 ± 0,5**	NS		**<0,0001**	**-6,7**
**AP overshoot (mV)**	**15,0 ± 1,2**	19,3 ± 1,2	**32,2 ± 1,4**	NS		**<0,0001**	**17,2**
**AP half-width (ms)**	**1,8 ± 0,06**	1,7 ± 0,04	**1,5 ± 0,04**	NS		**0,0356**	**-0,3**
**fAHP (mV)**	**-19,8 ± 0,6**	-18,4 ± 0,7	**-15,7 ± 0,7**	NS		**<0,05**	**-4,2**

Supplementary Table 2. Investigation of single-cell electrophysiological properties in control-derived and KO neuronal cultures.

Quantification of patch-clamp recordings of 10 weeks old control-derived, FAT4 KO and DCHS1 KO neurons. Recordings were performed in current-clamp mode. Data are represented as mean ± SEM. Statistical significance was based on one-way ANOVA with Turkey's multiple comparison tests. Independent wells were analyzed. At least twenty-two (n=22) randomly chosen neurons were analyzed across three independent batches (N=3). The exact p-values of significant differences and n numbers are shown in the table. Source data are provided as a Source Data file for the parameters significantly different, which graphs are shown in the Fig. S3m-o.

	Ctrl 1 (n = 37)	Ctrl 2 (n = 23)	DCHS1 (n = 39)	p1	Δ1	p2	Δ2
V_{rest} (mV)	-60,5 ± 1,0	-55,3 ± 0,6	-62,5 ± 1,2	NS		<0,001	-7,2
C_N (pF)	49,6 ± 2,5	44,2 ± 1,5	50,9 ± 3,2	NS		NS	
**AP threshold (mV)**	**-39,7 ± 0,9**	**-39,9 ± 0,6**	**-44,9 ± 0,9**	**0,0009**	**-5,2**	**0,0077**	**-5,0**
**AP overshoot (mV)**	**11,9 ± 1,1**	**14,3 ± 1,2**	**27,0 ± 1,9**	**<0,0001**	**15,1**	**<0,0001**	**12,7**
**AP half-width (ms)**	**1,8 ± 0,05**	**1,7 ± 0,03**	**1,4 ± 0,06**	**<0,0001**	**-0,4**	**0,0029**	**-0,3**
fAHP (mV)	-20,8 ± 1,0	-19,3 ± 0,7	-19,3 ± 0,8	NS		NS	

Supplementary Table 3. Investigation of single-cell electrophysiological properties in control- and DCHS1-derived neuronal cultures.

Quantification of patch-clamp recordings of 10 weeks old control- and DCHS1-derived neurons. Recordings were performed in current-clamp mode. Data are represented as mean ± SEM. Statistical significance was based on one-way ANOVA with Turkey's multiple comparison tests. Independent wells were analyzed. At least twenty-three (n=23) randomly chosen neurons were analyzed across three independent batches (N=3). The exact p-values of significant differences and n numbers are shown in the table. Source data are provided as a Source Data file for the parameters significantly different, which graphs are shown in the Fig. 3d-f.

Individual	Gene	Zygosity	DNA	Protein	Exon	Domain	PH
D2	DCHS1	Homozygous	c.2543delC	p.Thr848Asnfs*30	6	CR 8	+
F1	FAT4	Heterozygous	c.12476G>T	p.Cys4159Phe	14	LGLD	+
	FAT4	Heterozygous	c.13193G>A	p.Cys4398Tyr	17		

Supplementary Table 4. Genetic mutations in DCHS1 and FAT4 patients.

Indication of the detailed genetic mutations carried by the individuals presented in this study.

D2 patient carrying mutation in the *DCHS1* gene; F1 patient carrying mutations in the *FAT4* gene

Antigen	Dilution	Vendor	Catalog no.
SOX2	1/500	Abcam	AB5603
DoubleCortin (DCX)	1/1500	Millipore	AB2253
NEUN	1/500	Millipore	MAB377
NFIA	1/500	Novus Biologicals	NBP1-81406
S100β	1/500	Millipore	S2532
TBR1	1/500	Abcam	Ab31940
SATB2	1/500	Abcam	Ab51502
CTIP2	1/500	Abcam	Ab18465
CALR	1/500	Swant	CR7697
CALB	1/500	Sigma Aldrich	C9848
GABA	1/500	Sigma Aldrich	A2052
SCN3A	1/300	Thermo Fisher	PA5-77724
GFP	1/1000	Aves Lab	GFP-1020
SYN1-2	1/300	Synaptic systems	106006
MAP2	1/500	Sigma Aldrich	M4403
GAD67	1/500	Sigma Aldrich	MAB5406
SCN8A	1/300	Abcam	Ab65166

Supplementary Table 5. List of antibodies used.

List of antibodies used in the current study. Information of dilutions, vendors and catalogue numbers are listed.

Cell line used for 2D culture

Name of cell line	Official name	Reprogrammed cell type	Reprogramming method	Donor sex	Donor age	Patient sympthoms	Isogenic lines
Ctrl 1	GL	skin fibroblasts	Episomal (mmRNAs)		3yr		
Ctrl 2	WTC11 (GM25256)	skin fibroblasts	Episomal (mmRNAs)	male	30yr		
Ctrl 3	HPS00076:409B2	skin fibroblasts	Episomal (mmRNAs)	female	36yr		yes
FAT4	F1 (Fat4 mutation c.12476G>T)	skin fibroblasts	Episomal (mmRNAs)	male	12yr	PH, Deaf, Renal hypoplasia, Hand anomalies, Tracheal anomalies, Skeletal dysplasia	
DCHS1	D2 (Dchs1 mutation c.2543delC)	skin fibroblasts	Episomal (mmRNAs)	male	7yr	PH, Deaf, Renal hypoplasia, Hand anomalies, Tracheal anomalies, Skeletal dysplasia	
FAT4 KO	HPS00076:409B2	skin fibroblasts	Episomal (mmRNAs)	female	36yr		yes
DCHS1 KO	HPS00076:409B2	skin fibroblasts	Episomal (mmRNAs)	female	36yr		yes

Cell lines used for 3D hCOs

Name of cell line	Official name	Reprogrammed cell type	Reprogramming method	Donor sex	Donor age	Patient sympthoms	Isogenic lines
Ctrl 1	HPS00076:409B2	fibroblasts	Episomal (mmRNAs)	female	36yr		yes
Ctrl 2	FOR0018	PBMCs	Episomal (mmRNAs)	female	39yr		
FAT4	F1 (Fat4 mutation c.12476G>T)	fibroblasts	Episomal (mmRNAs)	male	12yr	PH, Deaf, Renal hypoplasia, Hand anomalies, Tracheal anomalies, Skeletal dysplasia	
DCHS1	D2 (Dchs1 mutation c.2543delC)	fibroblasts	Episomal (mmRNAs)	male	7yr	PH, Deaf, Renal hypoplasia, Hand anomalies, Tracheal anomalies, Skeletal dysplasia	
FAT4 KO	HPS00076:409B2	fibroblasts	Episomal (mmRNAs)	female	36yr		yes
DCHS1 KO	HPS00076:409B2	fibroblasts	Episomal (mmRNAs)	female	36yr		yes

Supplementary Table 6. Cell lines used in this study.

Detailed information about the cell lines used in this study are reported here: Name of cell line, official name, reprogrammed cell type and method, donor sex and age, patient symptoms and isogenic lines. The upper table (a) refer to the cell lines used to generate 2D neuronal cultures, the lower table (b) refer to the cell lines used to generate 3D hCOs.
