# Supplementary material for: Neuronal hyperactivity in neurons derived from individuals with gray matter heterotopia
Source: Nat Commun. 2025 Feb 18;16:1737. doi: 10.1038/s41467-025-56998-1 (PMC11836124; doi:10.1038/s41467-025-56998-1)
Supplement: Supplementary file 2 — Description of Additional Supplementary Files [file 41467_2025_56998_MOESM2_ESM.pdf]

Description of Additional Supplementary Files

Supplementary data 1. Proteomic data of control-, patient-derived and KO hCOs (full lysate and synaptosomal fractions).

Supplementary data 2. Transcriptomic data of control- and patient-derived hCOs
