# Supplementary material for: Neuronal hyperactivity in neurons derived from individuals with gray matter heterotopia
Source: Nat Commun. 2025 Feb 18;16:1737. doi: 10.1038/s41467-025-56998-1 (PMC11836124; doi:10.1038/s41467-025-56998-1)
Supplement: Supplementary file 5 — Reporting Summary [file 41467_2025_56998_MOESM5_ESM.pdf]

nature portfolio

Corresponding author(s): silvia.cappello@bmc.med.lmu.de
eder@psych.mpg.de

Last updated by author(s): Jan 5, 2025

Reporting Summary

Nature Portfolio wishes to improve the reproducibility of the work that we publish. This form provides structure for consistency and transparency in reporting. For further information on Nature Portfolio policies, see our Editorial Policies and the Editorial Policy Checklist.

Statistics

For all statistical analyses, confirm that the following items are present in the figure legend, table legend, main text, or Methods section.

n/a	Confirmed	
☐	☒	The exact sample size (*n*) for each experimental group/condition, given as a discrete number and unit of measurement
☐	☒	A statement on whether measurements were taken from distinct samples or whether the same sample was measured repeatedly
☐	☒	The statistical test(s) used AND whether they are one- or two-sided<br>*Only common tests should be described solely by name; describe more complex techniques in the Methods section.*
☒	☐	A description of all covariates tested
☐	☒	A description of any assumptions or corrections, such as tests of normality and adjustment for multiple comparisons
☐	☒	A full description of the statistical parameters including central tendency (e.g. means) or other basic estimates (e.g. regression coefficient) AND variation (e.g. standard deviation) or associated estimates of uncertainty (e.g. confidence intervals)
☐	☒	For null hypothesis testing, the test statistic (e.g. *F*, *t*, *r*) with confidence intervals, effect sizes, degrees of freedom and *P* value noted<br>*Give P values as exact values whenever suitable.*
☒	☐	For Bayesian analysis, information on the choice of priors and Markov chain Monte Carlo settings
☒	☐	For hierarchical and complex designs, identification of the appropriate level for tests and full reporting of outcomes
☐	☒	Estimates of effect sizes (e.g. Cohen's *d*, Pearson's *r*), indicating how they were calculated

Our web collection on statistics for biologists contains articles on many of the points above.

Software and code

Policy information about availability of computer code

Data collection	Leica software Las X, Genomics Cell Ranger 2.0 software, MaxQuant software 1.6.17.0, Neurolucida software 2017.03.3, Multichannel Experimenter software, FitMaster software, Xcalibur software
Data analysis	Image J win64, Illustrator CC, R Studio 4.1.1, GraphPad Prism 8.4.3, STAR 2.7.3a, RSEM 1.3.3, R/bioconductor 4.0.3, DEseq2 1.28.1, Panther, Neurolucida Explorer 2017.02.9, FitMaster software, Igor Pro software, NeuroExplorer software, Offline SorterTM software, MaxQuant software 1.6.17.0, Mini Analysis Program 6.0.7.

For manuscripts utilizing custom algorithms or software that are central to the research but not yet described in published literature, software must be made available to editors and reviewers. We strongly encourage code deposition in a community repository (e.g. GitHub). See the Nature Portfolio guidelines for submitting code & software for further information.

Data

Policy information about availability of data

All manuscripts must include a data availability statement. This statement should provide the following information, where applicable:

- Accession codes, unique identifiers, or web links for publicly available datasets
- A description of any restrictions on data availability
- For clinical datasets or third party data, please ensure that the statement adheres to our policy

All data supporting the findings described in this manuscript are available in the article and in the Supplementary Information, and from the corresponding author

upon reasonable request.
The genomic datasets are available on the GEO database under accession number GSE220673. The proteomic datasets generated and analyzed during the current study are available on the ProteomeXchange Consortium via the PRIDE46 partner repository with the dataset identifier PXD038760 and 10.6019/PXD038760.

Research involving human participants, their data, or biological material

Policy information about studies with human participants or human data. See also policy information about sex, gender (identity/presentation), and sexual orientation and race, ethnicity and racism.

Reporting on sex and gender	All data are provided in supplemetary table 9<br>Since this is a rare disorder, we selected the 2 available patient cell lines.
Reporting on race, ethnicity, or other socially relevant groupings	sex and/or gender was not considered in the study design due to limited samples (rare disease)
Population characteristics	*Describe the covariate-relevant population characteristics of the human research participants (e.g. age, genotypic information, past and current diagnosis and treatment categories). If you filled out the behavioural & social sciences study design questions and have nothing to add here, write "See above."*
Recruitment	*Describe how participants were recruited. Outline any potential self-selection bias or other biases that may be present and how these are likely to impact results.*
Ethics oversight	*Identify the organization(s) that approved the study protocol.*

Note that full information on the approval of the study protocol must also be provided in the manuscript.

Field-specific reporting

Please select the one below that is the best fit for your research. If you are not sure, read the appropriate sections before making your selection.

☒ Life sciences ☐ Behavioural & social sciences ☐ Ecological, evolutionary & environmental sciences

For a reference copy of the document with all sections, see nature.com/documents/nr-reporting-summary-flat.pdf

Life sciences study design

All studies must disclose on these points even when the disclosure is negative.

Sample size	For organoids we applied the same sample size determination by following previous publications (e.g. Klaus et al 2019, Kyrousi et al., 2021)
Data exclusions	No data were excluded from the analysis
Replication	All experiments were reproduced at least 3 times independently. All attempts for replications were successful.
Randomization	We do not have experimental groups, only 2 mutations were available. For all the other data we did not perform any randomization
Blinding	The majority of the data are acquired in a blinded manner.

Reporting for specific materials, systems and methods

We require information from authors about some types of materials, experimental systems and methods used in many studies. Here, indicate whether each material, system or method listed is relevant to your study. If you are not sure if a list item applies to your research, read the appropriate section before selecting a response.

Materials & experimental systems

n/a	Involved in the study	
☐	☒	Antibodies
☐	☒	Eukaryotic cell lines
☒	☐	Palaeontology and archaeology
☒	☐	Animals and other organisms
☒	☐	Clinical data
☒	☐	Dual use research of concern
☒	☐	Plants

Methods

n/a	Involved in the study	
☒	☐	ChIP-seq
☐	☒	Flow cytometry
☒	☐	MRI-based neuroimaging

Antibodies

Antibodies used

All antibodies are commercially available antibodies commonly used is several other publications. Every antibody was then validated in the lab to exclude non-specific signals due to secondary antibodies. A full list of the antibodies used is found in the manuscript and here:
Antibodies
MAP2 1:500 Sigma Aldrich M4403
GABA 1:500 Sigma Aldrich A2052
SYN1-2 1:300 Synaptic systems 106006
SATB2 1:500 Abcam Ab51502
S100β 1:500 Millipore S2532
GFP 1:1000 Aves Lab GFP-1020
DoubleCortin (DCX) 1:1500 Millipore AB2253
NEUN 1:500 Millipore MAB377
TBR1 1:500 Abcam Ab31940
CTIP2 1:500 Abcam Ab18465
GFAP 1:500 DAKO Z0334
CALB 1:500 Sigma Aldrich C9848
CALR 1:500 Swant CR7697
NFIA 1:500 Novus Biologicals NBP1-81406
SCN3A 1:300 Thermo Fisher PA5-77724
SOX2 1:500 Abcam Ab5603
GAD67 1:500 Sigma Aldrich MAB5406
SCN8A 1:300 Abcam Ab65166

Validation

All antibodies that are commercially available were used according to manufactures instructions. Every antibody was validated in the lab to exclude non-specific signals due to secondary antibodies using as negative control sections (in the case of immunofluorescence) available in the lab.

Eukaryotic cell lines

Policy information about cell lines and Sex and Gender in Research

Cell line source(s)

Commercially available control fibroblasts which were reprogrammed in the lab to iPSCs were included in this study (ATCC)

Authentication

The iPSC line used in this study was genotyped (Klaus et al., 2019, Di Matteo et al., 2020, Miyaoka et al., 2014).

Mycoplasma contamination

All cells were tested for mycoplasma monthly. All cell used were tested negative.

Commonly misidentified lines
(See ICLAC register)

No commonly misidentified lines were used in this study

Plants

Seed stocks

Report on the source of all seed stocks or other plant material used. If applicable, state the seed stock centre and catalogue number. If plant specimens were collected from the field, describe the collection location, date and sampling procedures.

Novel plant genotypes

Describe the methods by which all novel plant genotypes were produced. This includes those generated by transgenic approaches, gene editing, chemical/radiation-based mutagenesis and hybridization. For transgenic lines, describe the transformation method, the number of independent lines analyzed and the generation upon which experiments were performed. For gene-edited lines, describe the editor used, the endogenous sequence targeted for editing, the targeting guide RNA sequence (if applicable) and how the editor was applied.

Authentication

Describe any authentication procedures for each seed stock used or novel genotype generated. Describe any experiments used to assess the effect of a mutation and, where applicable, how potential secondary effects (e.g. second site T-DNA insertions, mosiacism, off-target gene editing) were examined.

Flow Cytometry

Plots

Confirm that:

☒ The axis labels state the marker and fluorochrome used (e.g. CD4-FITC).

☒ The axis scales are clearly visible. Include numbers along axes only for bottom left plot of group (a 'group' is an analysis of identical markers).

☒ All plots are contour plots with outliers or pseudocolor plots.

☒ A numerical value for number of cells or percentage (with statistics) is provided.

Methodology

Sample preparation	Samples were collected from 5 organoids. Full experimental description of the sample preparation is included in the materials and methods section of the manuscript.
Instrument	FACS analysis was performed at a FACS Aria (BD) in BD FACS Flow TM medium, with a nozzle diameter of 100 µm
Software	Flow Jo
Cell population abundance	*Describe the abundance of the relevant cell populations within post-sort fractions, providing details on the purity of the samples and how it was determined.*
Gating strategy	Debris and aggregated cells were gated out by forward scatter, sideward scatter; single cells were gated out by FSC-W/FSC-A. Gating for fluorophores was done using samples stained with secondary antibody only. Flow rate was below 500 events/sec

☐ Tick this box to confirm that a figure exemplifying the gating strategy is provided in the Supplementary Information.
